# Chyle Leak after Right Axillary Lymph Node Dissection in a Patient with Breast Cancer

**DOI:** 10.1155/2021/8812315

**Published:** 2021-02-11

**Authors:** Ankita Sarawagi, Jessica Maxwell

**Affiliations:** University of Nebraska Medical Center, Department of Surgery, Division of Surgical Oncology, Omaha, NE 68198-6880, USA

## Abstract

**Background:**

A female patient was diagnosed with a right-sided chyle leak following right skin sparing mastectomy, axillary lymph node dissection, and immediate tissue expander placement in the setting of invasive ductal carcinoma status post neoadjuvant chemotherapy. *Summary*. Our patient underwent a level I and II right axillary lymph node dissection followed by an axillary drain placement. On the first postoperative day, a change from serosanguinous to milky fluid in this drain was noted. The patient was diagnosed with a chyle leak based on the milky appearance and elevated triglyceride levels in the fluid. While chyle leaks are rare after an axillary dissection and even rarer to present on the right side, it is a complication of which breast surgeons should be aware. The cause of this complication is thought to be due to injury of the main thoracic duct, its branches, the subclavian duct, or its tributaries. Management is usually conservative; however, awareness of this potential complication even on the right side is of the utmost importance

**Conclusion:**

Chyle leaks are an uncommon complication of axillary node dissections and even rarer for them to present on the right side. It can be diagnosed by monitoring the drainage for changes in appearance and volume and by conducting supporting laboratory tests. Conservative management is generally suggested.

## 1. Introduction

Postoperative chyle leak in the setting of breast cancer and surgery is an exceedingly rare complication of axillary lymph node dissection. It has a reported incidence rate of 0.36-0.84%, and the majority of the cases published report a leak on the left side with few previous reports of a chyle leak following a right-sided axillary dissection [1–4].

We present a case of chyle leak following right skin sparing mastectomy, axillary lymph node dissection, and immediate tissue expander placement in a patient with right breast invasive ductal carcinoma status post neoadjuvant chemotherapy.

## 2. Case Report

A 57-year-old female patient presented with the diagnosis of cT3N1 right breast invasive ductal carcinoma. No distant metastatic disease was noted on staging imaging. After completing neoadjuvant chemotherapy, the patient underwent a right skin-sparing mastectomy, sentinel lymph node biopsy and targeted axillary lymph node dissection, and immediate stage I breast reconstruction with tissue expander placement. Intraoperatively, 1 of 3 axillary lymph nodes was positive for malignancy on frozen section and a level I and II right axillary lymph node dissection was completed. Multiple bulky and soft nodes were found in the axilla. The wound was irrigated, and no bleeding or lymphatic leakage was noted. The remainder of the operation was completed without complications. One drain was placed in the right axillary dissection region, and two additional drains were placed at the right breast following the tissue expander placement.

On the first postoperative day, all three drains were found to have serosanguinous drainage at 12 hours postoperatively. At approximately 18 hours postoperatively, the patient was noted to have 100 CC of milky fluid in the right axillary drain. The differential diagnosis for the milky drainage included lymphatic obstruction due to the bulky lymph nodes found at the time of dissection and a right-sided chyle leak. Triglyceride level was sent on the drainage and returned elevated at 1,425 mg/dl. Drain fluid triglyceride levels > 100 mg/dl confirm the diagnosis of chyle leak [5]. The patient was managed in a conservative fashion with pressure dressings to the right axilla. She was instructed to follow a low-fat diet and discharged home on postoperative day 2.

The patient followed up in clinic on postoperative day 12. She reported that axillary drain fluid turned serous and decreased in output, approximately 50 cc/day. It was recommended that the patient continue conservative management with the drain in place and pressure dressing. One week later, the patient reported 27 cc of serous drainage in the past 24 hours. The patient was recommended to discontinue the pressure dressing at this time. The patient presented two days later with scant serous discharge in her drain. The drain was removed on postoperative day 20.

## 3. Discussion

The thoracic duct is the structure responsible for lymph drainage for most of the body. It rises superiorly from the cisterna chyli, ascends through the aortic hiatus of the diaphragm, crosses over the midline to the left side of the thorax at the level of the aortic arch, ascends above the left clavicle, and finally descends to empty into the junction of the left subclavian and internal jugular veins [6]. Many anatomic variations have been described, including branches to the right jugular or subclavian veins [6]. A chyle leak during the postoperative period after head and neck surgery is a known phenomenon. For example, this risk following surgery for multinodular goiter ranges from 0.5 to 2% [7]. It is thought to be caused by injury to the thoracic duct and thus commonly presents on the left side [1]. The same phenomenon after axillary node dissection or left sided sentinel node in the setting of breast cancer is very rare. While not completely understood, there seem to be two main hypotheses on how this occurs: injury to an aberrant branch of the thoracic duct and chyle reflux due to injury of the subclavian duct [4]. The typical anatomy as outlined above is only present in about 50% of individuals. Some of the anatomical variants include termination of the thoracic duct into the external jugular vein, vertebral vein, brachiocephalic vein, suprascapular vein, and transverse cervical vein and terminating as a single vessel, bilateral ducts, or several terminal branches. Additionally, it has been shown that the duct empties on the right in 2-3% of cases and bilaterally in 1.5% of cases [6]. Chylous drainage may be noted postoperatively due to the presence of microscopic tributaries, specifically in the setting of occlusion due to a mediastinal mass. No mass was seen on staging imaging in this patient.

As the anatomical location of the thoracic duct is generally remote from the axilla, many use these variations to explain how injury to the duct while dissection might be possible. Taylor et al. conclude that an injury to an aberrant branch of the thoracic duct is possible during axillary node dissection and can present with a chyle leak [8].

Others, however, disagree that even an aberrant branch of the thoracic duct could be injured during axillary node dissection. Singh et al. postulate that injury to the main duct or its branches is not possible as variations to the normal anatomy of the thoracic duct occurs within 1 cm of the jugulovenous venous junction and an axillary node dissection does not extend to this level [9]. Instead, they propose that chyle reflux in the setting of this dissection occurs due to injury of the subclavian duct or a tributary [9].

In their case report, Daggett et al. present 37 known cases of chyle leak after axillary node dissection with only three presenting on the right side [4]. Daggett et al. reviewed the literature from 1993 to 2011, suggesting that this case is, to our knowledge, the first report of a right sided leak since their review.

Chyle leak following axillary node dissection most commonly presents on the first few days postoperatively, as was the case for this patient. It presents with drainage of a milky, nonpurulent fluid in the drain in place of serosanguinous or serous fluid [10, 11]. Laboratory testing is used to confirm the presence of a chyle leak using tests such as triglycerides, protein, cell count, lipoprotein electrophoresis, cholesterol, or pH [4]. Management requires adequate drainage and pressure dressings and can also include diet modifications to include a low-fat diet. Surgery is rarely required but may be necessary if the leak is identified intraoperatively or if it persists after conservative management [8]. Our patient responded favorably to conservative management.

It is standard practice to leave a drain in place following an axillary node dissection. This allows for monitoring of the quality of the output as well as avoidance of seroma formation. The duration of drain placement is surgeon dependent; however, it is our practice to remove axillary drains when serous output is <30 cc/day for two consecutive days. We recommend that in the rare incidence of chylous drainage, conservative management attempted for at least 3-4 weeks. This will likely require the drain to remain in place for a longer duration, and infection risk must be considered in this setting. Although the incidence of chyle leak is low in this commonly performed procedure, surgeons should be aware of this potential complication, even on the right side and how to manage it, and inform patients accordingly.

## 4. Conclusion

This case report represents a rare occurrence of right sided chyle leak following axillary node dissection. A chyle leak following an axillary node dissection is an unusual complication that generally occurs on the left side but has occasionally presented on the right. Though the axilla is remote from the site of the thoracic duct, injury to the thoracic duct, one of its multiple described aberrant branches, the subclavian duct, or its tributaries could cause this complication. Breast surgeons should be aware of further variation from normal anatomy including emptying of the thoracic duct on the right or bilaterally can explain the occurrence of a chyle leak on the right side, as well as how to identify and manage this complication.

## 5. Lessons Learned

A chyle leak is a rare but possible complication of axillary node dissection and even more unusual on the right side. Breast surgeons should be aware that variations of normal anatomy may explain the occurrence of a chyle leak on the right side. They should thus understand this possibility and how to identify and manage this complication.

## Data Availability

Data is available in the manuscript.
